# Evolutionary history and leaf succulence as explanations for medicinal use in aloes and the global popularity of *Aloe vera*

**DOI:** 10.1186/s12862-015-0291-7

**Published:** 2015-02-26

**Authors:** Olwen M Grace, Sven Buerki, Matthew RE Symonds, Félix Forest, Abraham E van Wyk, Gideon F Smith, Ronell R Klopper, Charlotte S Bjorå, Sophie Neale, Sebsebe Demissew, Monique SJ Simmonds, Nina Rønsted

**Affiliations:** Jodrell Laboratory, Royal Botanic Gardens, Kew, Surrey, London, TW9 3DS UK; Natural History Museum of Denmark, University of Copenhagen, Sølvgade 83 Entrance S, DK1307 Copenhagen K, Denmark; Department of Life Sciences, Natural History Museum, Cromwell Road, London, SW7 5BD UK; Centre for Integrative Ecology, School of Life & Environmental Sciences, Deakin University, 221 Burwood Highway, Burwood, Victoria 3125 Australia; Department of Plant Science, H.G.W.J. Schweickerdt Herbarium, University of Pretoria, Pretoria, 0002 South Africa; Biosystematics Research & Biodiversity Collections Division, South African National Biodiversity Institute, Private Bag X101, Pretoria 0001, South Africa; Department of Botany, Nelson Mandela Metropolitan University, PO Box 77000, Port Elizabeth, 6031 South Africa; Departamento de Ciências da Vida, Centre for Functional Ecology, Universidade de Coimbra, 3001-455 Coimbra, Portugal; Natural History Museum, University of Oslo, PO Box 1172, Blindern, NO-0318 Oslo Norway; Centre for Middle Eastern Plants, Royal Botanic Garden Edinburgh, 20A Inverleith Row, Edinburgh, EH3 5LR UK; Department of Plant Biology and Biodiversity Management, National Herbarium, College of Natural Sciences, Addis Ababa University, PO Box 3434, Addis Ababa, Ethiopia

**Keywords:** *Aloe vera*, Evolution, Biogeography, Phylogeny, Medicinal use, Succulent plants

## Abstract

**Background:**

*Aloe vera* supports a substantial global trade yet its wild origins, and explanations for its popularity over 500 related *Aloe* species in one of the world’s largest succulent groups, have remained uncertain. We developed an explicit phylogenetic framework to explore links between the rich traditions of medicinal use and leaf succulence in aloes.

**Results:**

The phylogenetic hypothesis clarifies the origins of *Aloe vera* to the Arabian Peninsula at the northernmost limits of the range for aloes. The genus *Aloe* originated in southern Africa ~16 million years ago and underwent two major radiations driven by different speciation processes, giving rise to the extraordinary diversity known today. Large, succulent leaves typical of medicinal aloes arose during the most recent diversification ~10 million years ago and are strongly correlated to the phylogeny and to the likelihood of a species being used for medicine. A significant, albeit weak, phylogenetic signal is evident in the medicinal uses of aloes, suggesting that the properties for which they are valued do not occur randomly across the branches of the phylogenetic tree.

**Conclusions:**

Phylogenetic investigation of plant use and leaf succulence among aloes has yielded new explanations for the extraordinary market dominance of *Aloe vera*. The industry preference for *Aloe vera* appears to be due to its proximity to important historic trade routes, and early introduction to trade and cultivation. Well-developed succulent leaf mesophyll tissue, an adaptive feature that likely contributed to the ecological success of the genus *Aloe*, is the main predictor for medicinal use among *Aloe* species, whereas evolutionary loss of succulence tends to be associated with losses of medicinal use. Phylogenetic analyses of plant use offer potential to understand patterns in the value of global plant diversity.

**Electronic supplementary material:**

The online version of this article (doi:10.1186/s12862-015-0291-7) contains supplementary material, which is available to authorized users.

## Background

The succulent leaf tissue of *Aloe vera* is a globally important commodity, with an estimated annual market of $13 billion [1]. The ‘gel’ tissue—polysaccharide-rich inner leaf mesophyll—provides a reservoir of water to sustain photosynthesis during droughts, and has been ascribed multiple bioactive properties associated with its use for skincare and digestive health [2]. *Aloe vera* has supported a thriving trade for thousands of years [3] and is arguably one of the most popular plants known in cultivation today, yet its origins in the wild have long been speculated. We have established that at least 25% of aloes (~120 species) are used for medicine yet fewer than 10 *Aloe* species are traded commercially, and these are used primarily for the purgative leaf exudate and on much lesser scales than *Aloe vera* (e.g. *Aloe ferox* in South Africa and *Aloe arborescens* in Asia) [4]. The immense market dominance of *Aloe vera* over other species of *Aloe* is not fully explained by available phytochemical evidence [5,6]. The extent to which the value of *Aloe vera* may be a consequence of evolutionary processes of selection and speciation, resulting in apparently unique properties and phylogenetic isolation, has not previously been considered.

*Aloe* (>500 species) is by far the most speciose of the six genera known collectively as aloes, which include *Aloiampelos* (7 species), *Aloidendron* (6 species), *Aristaloe* (1 species), *Gonialoe* (3 species) and *Kumara* (2 species). They are iconic in the African flora, and occur predominantly in eastern sub-Saharan Africa, and on the Arabian Peninsula, Madagascar and western Indian Ocean islands. Succulent plants are usually associated with arid environments; although numerous aloes occur in the drylands of Africa, they are also abundantly represented in tropical and subtropical vegetation infrequently impacted by drought. All aloes possess some degree of leaf succulence, as well as crassulacean acid metabolism (CAM) and a thick, waxy cuticle common in plants exhibiting a succulent syndrome [7]. Most are habitat specialists with narrow ranges and extraordinary rates of endemism, from an estimated 70% in southern Africa, 90% in Ethiopia, to 100% on Madagascar [8]. These centres of diversity coincide alarmingly with Africa’s biodiversity Hotspots, where a highly endemic biota is under substantial threat of extinction [9]. Risks posed by extensive habitat destruction and other threats to their survival are reflected by the inclusion of all aloes, except *Aloe vera*, in the Convention on International Trade in Endangered Species of Wild Fauna and Flora (CITES). The species-level diversity, ecological importance and threats to aloes place them among the world’s most important succulent plant lineages, other examples of which are ice plants (Aizoaceae), cacti (Cactaceae) and *Agave* (Agavaceae) [10]. Phylogenetic studies of related groups have focussed on the South African endemic *Haworthia* (e.g. [11,12]), whereas aloes have received little attention (but see [13]), and the origins and diversification of *Aloe* have remained unclear. It has therefore not been possible to determine whether *Aloe vera* is phylogenetically distinct from its many relatives, nor whether such phylogenetic distance may account for any potentially unique properties underpinning the value of the succulent leaf tissue.

Phylogenetic prediction is emerging as a promising tool for exploring correlations between the phylogenetic diversity and useful attributes of medicinal plants [14-17]. Rich biocultural traditions surround the use of aloe leaves for medicine, cosmetics, digestive health and general wellbeing [4]. Two natural products are derived from the leaves: carbohydrate-rich succulent leaf mesophyll tissue, applied topically to the skin or taken internally for digestion; and exudate, a liquid matrix high in phenolic compounds and most often used as a potent purgative, or in veterinary medicine (see [18]). The literature describing these uses is an untapped resource for understanding plant use in an evolutionary context, and in particular the extraordinary case of *Aloe vera*, which is used almost exclusively for its succulent leaf tissue. One point of interest is whether leaf succulence in aloes, which ranges from barely succulent in some species to very fleshy in others, could influence their use.

We aimed to explore the *Aloe vera* ‘phenomenon’ [5] by combining the largest ever phylogenetic hypothesis for the aloes with predictive methods. We used this to infer a scenario for their evolution, addressing a persistent gap in the understanding of global succulent plant diversity and biogeography. Links between the medicinal usefulness of aloes, their phylogenetic history, and extent of leaf succulence were evaluated by identifying evolutionary correlations and phylogenetic signal in uses and habit. Our comprehensive sampling represents the full morphological and geographical diversity of the aloes, and enabled the origins, geographical range evolution and divergence times of *Aloe* and relatives to be inferred. We synthesized our findings to determine whether the global value of *Aloe vera* can be better explained by evolutionary distinctiveness or by historical anthropogenic factors.

## Methods

### Phylogenetic hypothesis

A dataset was assembled representing seven plastid and nuclear DNA regions in 239 taxa in Xanthorrhoeaceae, including 197 species in the genera *Aloe*, *Aloidendron*, *Aloiampelos*, *Aristaloe*, *Gonialoe* and *Kumara*. We generated 480 new sequences from leaf or floral specimens collected from natural populations or from curated living collections and DNA banks held primarily at the Royal Botanic Gardens, Kew. A further 279 sequences were obtained from GenBank (ncbi.nlm.nih.gov/genbank/), including 93 *rbcL* and 64 *psbA* sequences. *Agapanthus africanus* (Amaryllidaceae) was used as the outgroup taxon in all analyses.

Total genomic DNA was isolated from fresh plant material (ca. 1 g) or specimens dried in silica gel (ca. 0.3 g) using a modified CTAB protocol [19] or the Qiagen DNeasy kit (Qiagen, Copenhagen). Sequences of ITS, *matK* and *trnL-F* were amplified using methodology previously described by [6]. The *trnQ-rps16* region was amplified with the primers trnQ(UUG)Aloe (5′-ATCTTRATACAATGTGATCCAC-3′; this study) and rps16x1 [20]. Sequences from the complementary strands were obtained for all taxa whenever possible, using the BigDye Terminator v3.1 on a 3730 DNA Analyzer (Applied Biosystems/Hitachi). Sequences were assembled in Sequencher 4.8 (Gene Codes, Ann Arbor) and submitted to GenBank (Additional file 1). Sequences were aligned automatically using MUSCLE [21] implemented with default settings in SeaView v4.2.12 [22], and adjusted manually in BioEdit v7.1.11 [23]. The DNA regions were aligned separately before the data were concatenated using an R [24] script to produce a final dataset comprising 240 taxa and 6732 nucleotides in seven DNA regions.

We used Bayesian inference, maximum likelihood and parsimony to produce a phylogenetic hypothesis for *Aloe* and allied genera*,* using single-partition (*ITS*, *matK*, *rps16*, *psbA*, *rbcL*, *trnL-F* intron and spacer) and combined datasets. We ran all analyses on the Cyber Infrastructure for Phylogenetic Research (CIPRES) portal [25]. Separate parsimony analyses of the ITS (175 taxa, 799 nucleotides) and plastid (231 taxa, 5933 nucleotides) datasets were undertaken with the parsimony ratchet implemented in PAUPRat [26], to check for strongly supported phylogenetic conflicts (bootstrap percentages >75), before proceeding with analyses based on a total evidence approach using all characters. A maximum likelihood analysis, comprising 1000 bootstrap replicates followed by a heuristic tree search, was executed in RAxML [27] with each partition assigned specific parameters under the recommended GTRCAT model. An additional 530 gaps and indels in the combined dataset of all DNA regions were coded using the algorithm described by [28] in the FastGap v1.2 interface [29]. Finally, we ran a Bayesian analysis of the combined dataset with gaps coded in MrBayes v3.1.2 [30]. Best-fitting models for each data partition for Bayesian inference were identified using the Akaike Information Criterion calculated in Modeltest v3.8 [31]. The Hasegawa, Kishino and Yano (HKY) model with gamma-shaped distribution of rate heterogeneity among sites (HKY + G) was selected for the ITS, *matK*, *trn*Q-*rps16* and *trnL-F* data partitions, while the General Time Reversible (GTR) model with gamma distribution of rate heterogeneity among sites was selected for *psbA* (GTR + G), and with a proportion of invariable sites (GTR + I + G) for *rbcL*. For the Bayesian analysis, the parameters were unlinked between loci and four Metropolis Coupled Markov Chains with heating increments of 0.2 were run for 50 million generations and sampled every 1000th generation. The resulting parameters were summarised in Tracer 1.5.0 [32]. A quarter of the least likely trees were discarded, and a majority rule consensus tree with branch supports expressed as posterior probabilities (PP) was produced from the remaining trees.

### Divergence time estimates and biogeographic scenario

Divergence times were estimated using a penalised likelihood (PL) approach previously applied in Hyacinthaceae, a related family in Asparagales, as described by [33]. In the absence of fossil data for aloes and related genera, analyses were constrained to the mean age of 34.2 Ma inferred for the crown node of Asphodeloideae in a recent study of all Asparagales families [34]. Due to the computational demands of analyses on the full Xanthorrhoeaceae dataset and our focus on the aloes (*Aloe*, *Aloiampelos*, *Aloidendron*, *Aristaloe*, *Gonialoe* and *Kumara*), we excluded subfamilies Xanthorrhoeoideae and Hemerocallidoideae from subsequent analyses and pruned the Bayesian consensus tree to 228 species in Asphodeloideae. The penalised likelihood method [35] was run on 1000 randomly selected trees from the Bayesian stationary distribution and summarised on the consensus tree [33]. The optimal rate smoothing value for this dataset was determined by cross validation on the pruned Bayesian consensus tree, using the Truncated Newton algorithm (S = 5) implemented in r8s v 1.8 [36]. The outgroup taxon was pruned prior to the estimation of divergence times, as required by r8s. Mean age values and 95% confidence intervals for the nodes on the Bayesian consensus tree were computed in TreeAnnotator [37].

A biogeographic scenario for the aloes was inferred using the dispersal-extinction-cladogenesis (DEC) likelihood model implemented in Lagrange v2.0.1 [38]. Species distribution data were compiled from authoritative checklists for Asphodeloideae [39,40] and standardised according to the Taxonomic Data Working Group (TDWG) guidelines [41]. We defined eight areas based on the statistically-delimited biogeographical regions of Africa, incorporating the faunal and floral diversity of the continent, described recently by [42]. For subfamily Asphodeloideae, Arabia, Madagascar and Eurasia were added to the Southern African, Zambezian and Congolian regions, together with expanded Ethiopian-Somalian and Saharan-Sudanian regions. Assigning species to areas was straightforward due to the typically narrow distribution of most *Aloe* species, and because neighbouring areas are separated by physical barriers or marked differences in climatic conditions. Ancestral area reconstructions in Lagrange [38] were performed on the dated consensus (allcompat) tree obtained from the penalised likelihood analysis. In brief, ancestral areas were computed at each node of the tree under the DEC likelihood model, following a method described in detail by [33]. Ancestral areas with a relative probability >1 were combined with the node age and lengths of the descendent branches on the tree to infer the frequency and nature of transition events between ancestral and descendant nodes [33]. The resulting biogeographic scenario was visualised on the dated Bayesian consensus tree using pie charts showing the likelihoods of all possible ancestral areas per node for subfamily Asphodeloideae.

### Phylogenetic signal in utility and habit

We interrogated a dataset of over 1400 use records from the literature [18] to investigate phylogenetic signal in the uses of aloes. Data were coded according to the Economic Botany Data Standard [43] from which two categories of use were considered. In the first category, we combined all TDWG Level 1 data to yield a discrete binary character describing any documented use, while the second comprised data in the TDWG Level 2 Medicines category. General use (e.g. for food, materials, social purposes, etc.) and medicinal use specifically were scored as present (=1) or absent (=0) in each of the terminal taxa. Records describing a plant as *not* used are unusual in the ethnobotanical literature, and hence in all cases 0 indicated a lack of reported use, rather than definitive knowledge of no use. The consensus (allcompat) tree inferred by Bayesian analysis with gaps coded was pruned to 197 species representing *Aloe*, *Aloiampelos*, *Aloidendron*, *Aristaloe*, *Gonialoe* and *Kumara.*

We calculated phylogenetic signal using the D metric [44], a measure specifically developed for quantifying phylogenetic signal in binary characters, implemented in the R package *caper* [45]. D compares the number of observed changes in a trait over a phylogeny with the number that would be expected under two alternative simulated scenarios: one where there is strong phylogenetic dependence and the trait has evolved via a gradual Brownian motion model of evolution, and the second where there is no phylogenetic dependence and the trait is randomly scattered across the species, regardless of phylogeny. The D metric generates a value that usually lies between 0 and 1, where a value of 1 indicates that the trait has evolved in essentially a random manner (i.e. no phylogenetic signal), and 0 indicates that the trait is highly correlated with phylogeny, in a manner predicted by Brownian motion. Tests for significant differences from D = 1 (no phylogenetic signal) are derived by simulating the random distribution of the trait among species 1000 times to generate a null distribution for the D statistic. We conducted the analysis in two ways, one using just the consensus phylogeny, and the second using 1000 trees selected at random from the Bayesian posterior distribution calculating median values for D and associated P values.

The putative contribution of leaf succulence to the ‘usefulness’ of aloes was explored using a phylogenetic comparative approach. A character set describing the extent of water-storing mesophyll tissue in the leaves was assembled from species descriptions [46-48] and observations of leaf morphology in aloes*.* Species were broadly scored as ‘succulent’ or ‘barely succulent’ and additionally classified as barely succulent shrubs (the grass aloes, *Aloe* section *Leptaloe*), succulent shrubs (*Aristaloe*, *Gonialoe* and most of *Aloe*), branching trees (*Aloidendron*, *Kumara*) and scrambling shrubs with variably succulent leaves (*Aloiampelos*). These were visualised on the Bayesian consensus tree by reconstructing the ancestral states of three characters (succulence, habit and medicinal use), scored as binary traits, under the parsimony optimisation in Mesquite [49].

For calculation of phylogenetic signal using the D metric in these traits, they were coded as four separate dummy binary variables (e.g. succulence: 0 = no, 1 = yes). Pairwise comparison tests [50] were used to assess possible evolutionary correlations between habit and documented uses generally and medicinal uses specifically (dependent variables). This method takes phylogenetically independent pairs of species and observes any correlated differences in the states of two binary characters. For every gain or loss in one character (in this case, the measure of leaf succulence), it assesses whether there is an associated loss, gain or no change in the other (medicinal or general use), and compares any patterns with those expected if the second character were randomly distributed on the phylogeny. Pairwise comparison calculations were carried out using Mesquite [49]. As with our D metric calculations, to account for uncertainty in the phylogenetic topology and weak branch supports, we ran all the analyses on the Bayesian consensus (allcompat) topology (using 100 randomly selected sets of pairwise comparisons) and on a random sample of 1000 trees from the Bayesian posterior distribution, calculating median probability values associated with the correlation.

## Results

### Phylogenetic hypothesis

**Figure 1:**
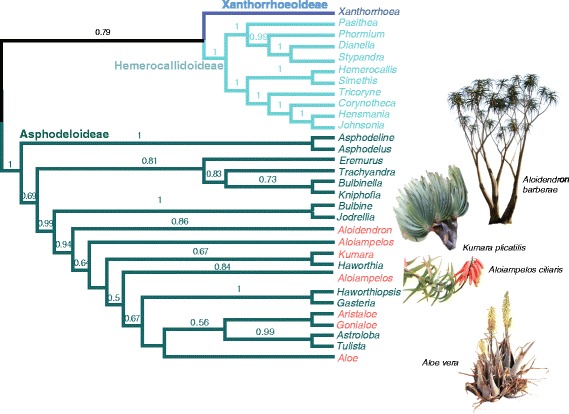
**Subfamilies and genera of Xanthorrhoeaceae.** Summary phylogram with Bayesian posterior probabilities (>0.5) above branches; red branches represent the six genera known collectively as aloes: *Aloe*, *Aristaloe*, *Gonialoe*, *Kumara*, *Aloiampelos* and *Aloidendron*.

Our phylogenetic analyses of >7 kb plastid and nuclear characters (6732 nucleotides and 550 gaps) in ca. 40% of *Aloe* species substantiate current understanding of taxonomic relationships in Xanthorrhoeaceae subfamily Asphodeloideae [11,12,51] and divergence times within Asparagales [33] (Figure 1, Additional files 2 and 3). We sampled 26 genera and 240 species in Xanthorrhoeaceae, using a total evidence approach despite sequence data for some taxa being incomplete (Additional files 1 and 4). The effects of missing data on phylogenetic analyses have been widely debated, but there is convincing evidence for the accurate phylogenetic placement of taxa with considerable missing data (summarised by [52]). Model-based methods of phylogenetic inference perform better than parsimony in estimating trees from datasets with missing data [53,54], and we therefore based subsequent analyses on the Bayesian phylogenetic inference (Additional file 3). Low levels of genetic polymorphisms, taxonomic complexities, and the number of inaccessible, narrowly distributed species challenge the study of aloes; this is the first phylogeny to include >10% of *Aloe* species*.* Parsimony and maximum likelihood topologies (trees not shown) compared well to the Bayesian tree used in downstream analyses. Branching tree aloes (*Aloidendron*) are basal to the remainder of the alooids. A clade comprising the Cape endemic genus *Kumara* and *Haworthia s.s.* is sister to *Aloiampelos*, which is in turn sister to *Aloe*. Within the large *Aloe* clade (184 species), well-supported terminal branches highlight species-level relationships but the clades, which will ultimately underpin a taxonomic revision, are incompletely resolved. The placement of *Aloiampelos juddii* at the base of the alooid topology, on a branch sister to *Kumara-Haworthia*, warrants further investigation of reciprocal monophyly in *Aloiampelos*. We included four members of *Astroloba*, two *Tulista*, three *Haworthiopsis* and four *Haworthia* in our study and recovered these as paraphyletic with varying support. The haworthioid taxa were, until recently [12], phylogenetically problematic (e.g. [11]).

**Figure 2:**
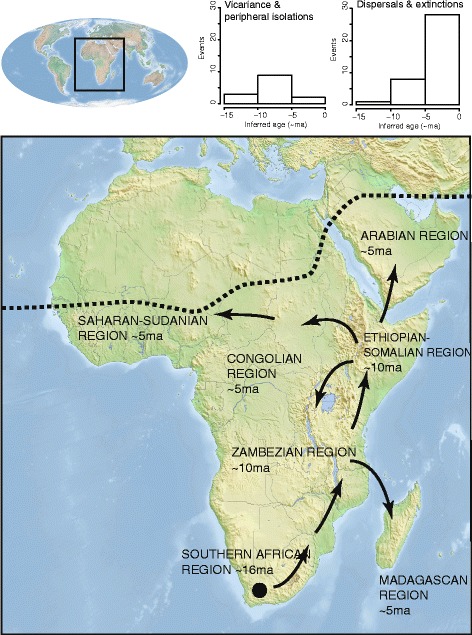
**Biogeographic scenario for** ***Aloe.*** Distribution and biogeographic scenario for *Aloe* inferred from nucleotide and plastid data for 228 taxa in Xanthorrhoeaceae subfamily Asphodeloideae. Enlarged map shows the natural distribution of *Aloe*, with northernmost limits indicated by dashed line. Direction and timing of diversification events inferred from ancestral state reconstruction and penalised likelihood dating are shown by arrows. Histograms show branch-based (dispersal and extinction) and node-based (vicariance and peripheral isolations) events in speciation processes since the divergence of the *Aloe* crown group ~16 Ma.

**Figure 3:**
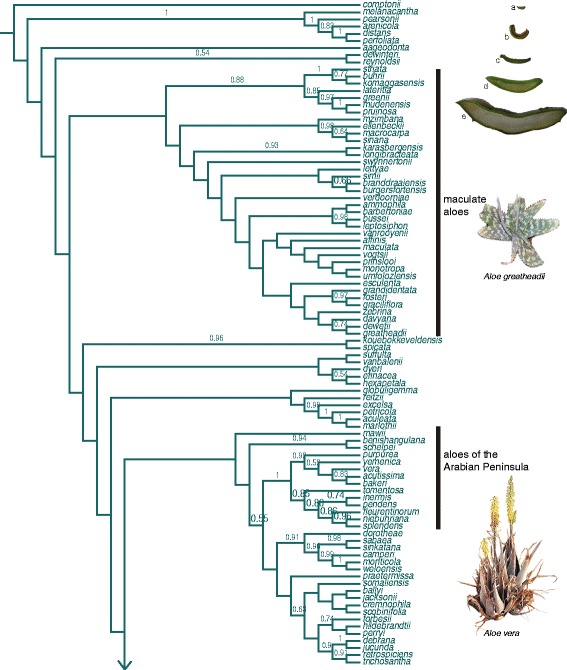
**Bayesian consensus tree for** ***Aloe.*** Core *Aloe* clade from a Bayesian analysis of Xanthorrhoeaceae highlighting relationships of interest in the biogeographical scenario*.* Inset shows representative variation in the extent of leaf succulence among aloes: a, *Aloiampelos ciliaris*; b, *Aloidendron eminens*; c, *Kumara plicatilis*; d, *Aloe vera*; e, *Aloe marlothii.*

**Figure 4:**
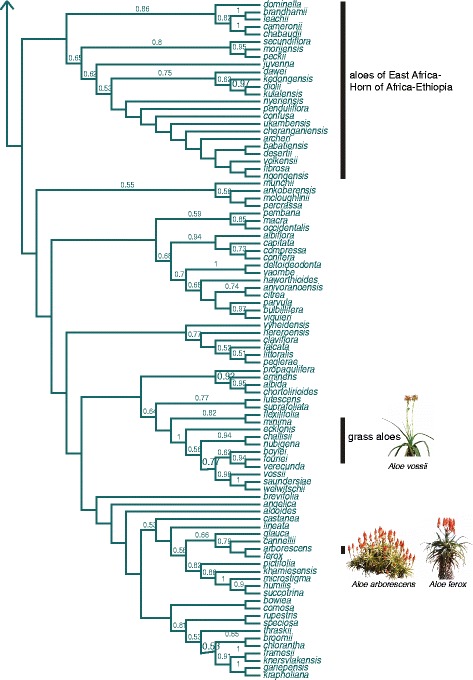
**Bayesian consensus tree for** ***Aloe*** **(continued from Figure** 3 **).**

### Divergence time estimates and biogeographic scenario

Divergence times estimated using a penalised likelihood approach and ancestral area reconstructions revealed that aloes originated in southern Africa in the early Miocene, ~19 million years ago (Ma) (Additional file 3). *Aloe vera* was recovered in a strongly supported clade with eight other Arabian species, allowing us to infer its origins on the Arabian Peninsula within the last five million years. Two southern African species supporting commercial natural products industries, *Aloe arborescens* and *A. ferox*, were recovered together in a southern African clade. We estimate that the diversification of the genus *Aloe* began ~16 Ma in South Africa with a period of range expansion of ancestral taxa north-eastwards into the Zambezian and Ethiopian-Somalian regions ~10 Ma. Peripheral isolation and, to a lesser extent, vicariance were inferred to be the major speciation processes for the early diversification of aloes until around ~5 Ma, when a sharp increase in dispersal events occurred in several near-simultaneous radiations of the aloes at the extremities of their range, particularly in Madagascar. During this period, aloes reached West Africa, the Saharan-Sudanian region and the Arabian Peninsula via the Ethiopian-Somalian region, and arrived on Madagascar from the Zambezian region (Figure 2). This scenario identifies the Ethiopian-Somalian region as a cross-road for speciation processes in *Aloe*, as the majority of dispersal events (16 events) in our dataset were from here into each of the four adjacent regions. We identified multiple introductions to Madagascar (three dispersals). Similarly, diversification of aloes on the Arabian Peninsula resulted from one or more dispersals, as well as vicariance and peripheral isolation, with no evidence of dispersal back to continental Africa. A single southerly dispersal event was detected from the Zambezian to the Southern African regions.

### Phylogenetic signal in utility and habit

Leaf succulence increased steadily with the emergence of aloes in southern Africa, from the barely succulent tree aloes (*Aloidendron* and *Kumara*) and rambling aloes *(Aloiampelos*), to *Aloe* and neighbouring genera (Figures 1, 3 and 4). Though difficult to quantify, pronounced succulence is restricted to *Aloe*, and has been almost completely lost in several members of this genus, notably in the clade comprising southern African grass aloes, *Aloe* section *Leptaloe*, during the last ~10 Ma (Figure 3; Additional file 5 a-c). The habit of relatively large, succulent leaves borne in basal rosettes on an unbranched stem, typical of *Aloe vera* and other commercially valuable species, exhibits a strong phylogenetic signal. Using Fritz & Purvis’s D-metric [44] as our measure of phylogenetic signal, where D = 1 indicates no phylogenetic structure to the trait data and D = 0 indicates strong correlation between trait distribution and phylogeny (see methods for full description), we found the degree of phylogenetic signal in succulence *per se* was highly significant (D = 0.132, p < 0.001).

Uses are documented for 48% of the aloes sampled in this study*.* Of the 81 *Aloe* species in our analysis that have documented medicinal use, 98% have succulent leaves. By contrast, in 87% of the 15 species in which succulent leaf mesophyll has been almost entirely lost, there is negligible documented tradition of medicinal use, even in regions with thoroughly documented ethnoflora, such as South Africa. Whilst many succulent-leaved aloes do not have known medicinal uses, the likelihood of use is significantly higher in the succulent aloes (Fisher’s exact test comparing proportions of succulent vs. non-succulent species with medicinal utility, prior to considering phylogenetic effects: p = 0.014). Our pairwise comparison analyses indicated that there most likely have been six evolutionary losses of leaf succulence in aloes, as predicted from the Bayesian consensus phylogeny and 884 of the 1000 Bayesian posterior distribution trees. With the consensus tree, we tested the hypothesis that the use of an aloe for medicine diminishes or is lost entirely with a reduction in leaf succulence. Four of the six evolutionary transitions in aloes where succulence is severely reduced are associated with loss of medicinal use, providing weakly significant support for the hypothesis (pairwise comparison test: p = 0.065). The same analysis using 1000 trees sampled randomly from the Bayesian posterior distribution, was unable to resolve clearly whether four or three transitions in aloe leaf succulence were associated with loss of medicinal use (p = 0.125). We detected a weak phylogenetic signal in the general use of these genera (D = 0.828, p = 0.063). Focussing on the use of aloes for medicine, we also identified a weak, but significant, phylogenetic signal (D = 0.794, p = 0.029).

Species with documented medicinal use are not randomly distributed across the phylogeny. In a large clade comprising 29 species of maculate aloes, characterised by large, succulent leaves and a short stem, 55% are used for medicine. A clade of 18 closely related species native to East Africa, Ethiopia and the Horn of Africa (the Zambezian and Ethiopian-Somalian biogeographic regions) included 27% species with known medicinal uses. *Aloe vera* was among three medicinal species in a clade of eight species native to the Arabian Peninsula.

## Discussion

The phylogenetic hypothesis for the aloes reveals a distinctive geographical pattern of major clades of *Aloe* with four biogeographical centres of diversity: Southern Africa (~170 species), Madagascar (~120 species), East Africa/Zambezian region (~100 species), and the Horn of Africa/Ethiopian-Somalian region (~90 species). Based on strongly supported close relationships with morphologically similar Arabian species, we clarify that *Aloe vera* is native to the Arabian Peninsula. Previous suggestions have included Sudan or the Arabian Peninsula, based on morphological affinities with Arabian species [55] and even further afield in the Canary Islands, Cape Verde Islands, Madeira or Spain [3], which could be explained by naturalised populations, introduced via ancient trade routes, being mistaken for indigenous elements of the flora. Our explicitly phylogenetic context places *Aloe vera* for the first time among related Arabian species at the northernmost natural range limit of aloes, in habitats at the extremes for aloes in terms of aridity and diurnal temperature fluctuations. Here, at the hot and dry edge of their natural range, aloes are characterised by leathery, glaucous leaves that likely protect the water-storing leaf mesophyll from diurnal temperature and radiation extremes. The evolutionary distinctiveness of aloes on the Arabian Peninsula which could account for atypical properties in *Aloe vera*, is thrown into question by their affinities with species in the Ethiopian-Somalian region [13,56]. We found evidence for at least one dispersal from the Ethiopian-Somalian region to the Arabian Peninsula within the last 5 Ma. The biogeographic scenario inferred here (Figure 2) elucidates the diversification of *Aloe* prior to its arrival in north-east Africa and the Arabian Peninsula, and reveals a southern African cradle for the genus ~16 Ma, in the early Miocene. Consequently, the longstanding hypothesis that aloes first appeared in southeast Africa considerably earlier, in the late Mesozoic-early Cenozoic [56] is contradicted by the molecular evidence in the present study.

The establishment of the Mediterranean climate in south-western Africa and the expansion of southern African deserts in the Miocene caused large-scale extinctions in the prevailing subtropical flora [57] and appear to have had a profound impact on the evolution of aloes. Habitat expansion has been proposed as the main driver for the simultaneous global diversification of plants with a succulent habit [10]. But on a local scale, loss of suitable habitat forced southern African aloes to migrate north-eastwards as these species struggled to adapt to bioclimatic changes at the southernmost tip of Africa. The establishment of the winter-rainfall region, in particular, appears to have largely excluded aloes from the semi-arid Succulent Karoo region, a celebrated global centre of succulent plant diversity with ~5,000 species and 40% endemism. The Succulent Karoo flora is characterised by short-lived, drought-sensitive dwarf and leaf-succulent shrubs [58] such as the ~1500 members of Aizoaceae subfamily Ruschioideae [59] and ~1000 species of Crassulaceae [60]. Aloes, in contrast, tend to be long-lived and drought tolerant, and are relatively poorly represented in the Succulent Karoo and winter-rainfall regions of southern Africa. Water-use efficiencies may have placed even the earliest, barely-succulent aloes at an ecological advantage over non-succulent lineages in the relictual subtropical vegetation.

The timing of two periods of diversification detected in aloes, in the late Miocene and more recently in the Pliocene, coincide remarkably with the simultaneous ‘burst’ of evolution in major succulent plant lineages globally, attributed to a rapid decline in atmospheric CO_2_ and increased aridity during the mid- to late Miocene [10]. Consistently low rates of extinction in our data agree with previous findings [56], suggesting continuous but irregular diversification of aloes. We identified a distinct shift from node-based speciation processes (namely, vicariance and peripheral isolations) to branch-based events (dispersals and extinctions) coincident with each of the radiations of the aloes*.* We interpret this as a period of range expansion and diversification of relatively widespread species until the Miocene-Pliocene boundary. The second rapid diversification was likely the result of species fragmentation and increased niche availability, when isolated taxa dispersed short distances into the rich habitat mosaics formed by geological processes during the Pliocene, giving rise to the present-day distribution of *Aloe*. This is evident in a five-fold increase in dispersal events in our dataset, while node-based processes and extinctions are low to negligible during the same period. The tempo of these pulsed radiations in *Aloe* is strikingly similar to that of *Agave* (Agavaceae), a New World group of ~200 species of leaf succulent rosette plants [61], adding new depth to this celebrated example of convergent evolution among succulents.

The evolution of leaf succulence followed the pattern of divergence in aloe and relatives, in tandem with the expansion of semi-arid habitats in Africa between ~ 10 and 5 Ma. Like the earliest cacti [62], ancestral aloes were barely succulent and tree-like. Large and markedly succulent leaves are restricted to the genus *Aloe* and, unlike other lineages in which succulence has arisen multiple times (e.g. Portulacineae [63] and Aizoaceae [59]), variation in the extent of leaf succulence among species of *Aloe* is due to loss of water-storing tissues (e.g. in the barely succulent grass aloes) (Figure 3). The idea that rich traditions of use in the aloes may be linked to the extent of leaf succulence has not been previously investigated, and our analyses suggest that a decrease in the proportion of water-storing leaf mesophyll reduces the possibility that a species is used for medicine, irrespective of whether the leaf mesophyll tissue and/or liquid exudate are used. Documented medicinal uses for barely succulent members of *Aloidendron*, *Kumara* and *Aloiampelos* focus on the roots or leaf exudate, and never the leaf mesophyll. Additionally, our phylogenetic reconstruction suggests that medicinal utility appears less likely in lineages where reduced succulence has evolved. For instance, we found very few documented medicinal uses for the barely-succulent grass aloes despite their relative abundance in regions with thoroughly documented ethnoflora, such as the fire-adapted grasslands of KwaZulu-Natal in South Africa. We detected weak, but significant, phylogenetic signals in the use of aloes generally, and for medicinal purposes specifically. A comparable study of the Amaryllidaceae, a family with well-characterised bioactive alkaloids, recovered a similar overall phylogenetic signal for medicinal use [15].

A link between leaf succulence and medicinal use suggests a traditionally pragmatic approach to the selection of aloes with large, succulent leaves for use in medicine [4]. Features such as firm leaf mesophyll, a short stem, small teeth on the leaf margins, and ease of propagation, are shared by *Aloe vera* and numerous other *Aloe* species used medicinally, including closely related species from the Arabian Peninsula and the Ethiopian-Somalian region. Our evolutionary hypothesis for *Aloe* locates *Aloe vera* in close phylogenetic proximity to seven other species native to the Arabian Peninsula, discounting a distinctive evolutionary history for *Aloe vera* which could imply unique leaf properties. Mounting anecdotal evidence for the beneficial properties of *Aloe vera* continues to stimulate research into the bioactivity of the succulent leaf mesophyll [2]. Recent studies of *Aloe vera* and a phylogenetically-representative sampling of nearly 30 *Aloe* species have shown very low levels of variation in the monosaccharide composition of leaf mesophyll carbohydrates [6,64], although differences in carbohydrate structure may yet be discovered pending systematic evaluation of these highly complex carbohydrates which are assumed to be responsible for the medicinal value of the leaf mesophyll [5]. On the other hand, documented traditions of use indicate that few of the closest relatives of *Aloe vera* are used medicinally. Records of the therapeutic uses of *Aloe vera* leaf mesophyll and exudate date to classical times [3,5,65]. Trade routes for *Aloe vera* were well established in the Red Sea and Mediterranean by the 4th century BCE [3] and, assuming the species occupied a narrow range typical of Arabian aloes, it may have been rapidly harvested to near-extinction to meet market demands. The remarkable contemporary market dominance of *Aloe vera* over other aloes therefore appears to be the consequence of its origins near important early trade routes, ancient selection for medicine and cultural history, which introduced the species into trade and cultivation thousands of years ago.

## Conclusion

Phylogenetic investigation of plant use and leaf succulence among aloes has yielded new explanations for the extraordinary market dominance of *Aloe vera*. The evolutionary history inferred from our analyses of *Aloe* and related genera shows for the first time that *Aloe vera* is native to the Arabian Peninsula, and discounts phylogenetic distance as an explanation for its popularity over many other species of *Aloe*. The industry preference for *Aloe vera* appears to be due to its proximity to important historic trade routes, and early introduction to trade and cultivation. Well-developed succulent leaf mesophyll tissue, an adaptive feature that likely contributed to the ecological success of the genus *Aloe*, is the main predictor for medicinal use among *Aloe* species, whereas evolutionary losses of succulence tend to be associated with losses of medicinal use. Phylogenetic analyses of plant use offer potential to understand patterns in the value of global plant diversity.

### Data accessibility

DNA sequences are deposited in GenBank and accession numbers are listed in Additional file 1.

The phylogenetic tree supporting the results of this article is available in the TreeBase repository, http://purl.org/phylo/treebase/phylows/study/TB2:S16954?format=html [66].

## Additional files

Additional file 1:
**GenBank sequence data for taxa studied.** Accession/collectors’ numbers and international codes for herbaria where vouchers are deposited are given for sequences from this study: C, Copenhagen; E, Edinburgh; ETH, Addis Ababa; K, Kew; O, Oslo; NBG, Compton; PRE, Pretoria; DNA signifies DNA bank accession; — signifies no sequence. Additional file 2:
**Phylogenetic hypothesis for Xanthorrhoeaceae.** Bayesian consensus tree for 240 species of Xanthorrhoeaceae subfamilies Xanthorrhoeoideae, Hemerocallidoideae and Asphodeloideae, with posterior probabilities >0.5 displayed above branches. Additional file 3:
**Ancestral area reconstructions for Xanthorrhoeaceae subfamily Asphodeloideae.** a) Ancestral areas displayed on the penalised likelihood-dated Bayesian consensus tree; b) detail of the clade containing *Aloe vera*. Legend refers to regions modified from [57] for this analysis: A, Southern Africa; B, Zambezi; C, Congolian; D, Ethiopian-Somalian; E, Saharan-Sudanian; F, Arabian; G, Madagascan; H, Eurasian; Trash, sum of ancestral area probabilities <0.1. Additional file 4:
**Summary statistics for phylogenetic dataset.** Taxon sampling, sequence length and model selection for data partitions. Additional file 5:
**Phylogenetic distribution of leaf succulence, habit and medicinal use in alooid taxa.** Most parsimonious reconstructions of character states mapped to Bayesian consensus tree in a) leaf succulence, b) habit and c) medicinal uses.
