# Supplementary figures and images for: Evolutionary history and leaf succulence as explanations for medicinal use in aloes and the global popularity of Aloe vera
Source: BMC Evol Biol. 2015 Feb 26;15:29. doi: 10.1186/s12862-015-0291-7 (PMC4342203; doi:10.1186/s12862-015-0291-7)

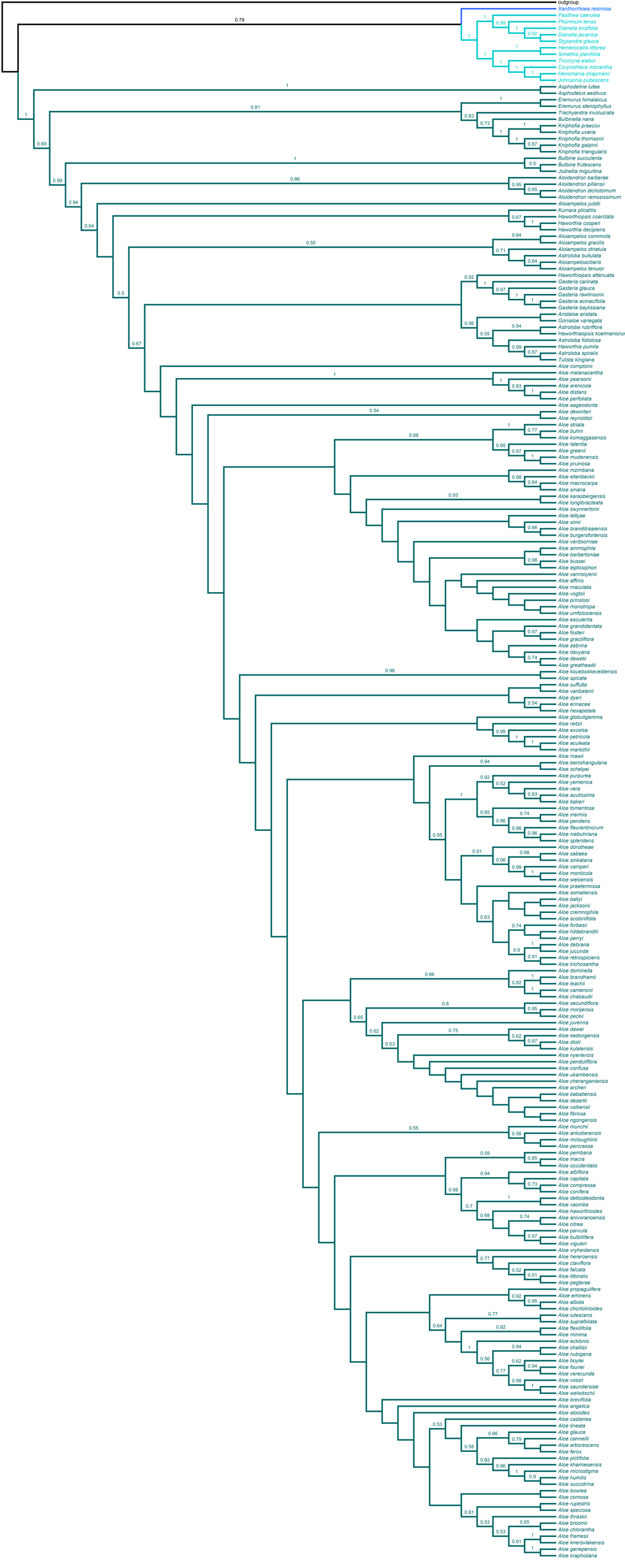

Supplement: Additional file 2: — Phylogenetic hypothesis for Xanthorrhoeaceae. Bayesian consensus tree for 240 species of Xanthorrhoeaceae subfamilies Xanthorrhoeoideae, Hemerocallidoideae and Asphodeloideae, with posterior probabilities >0.5 displayed above branches. [file 12862_2015_291_MOESM2_ESM.pdf]

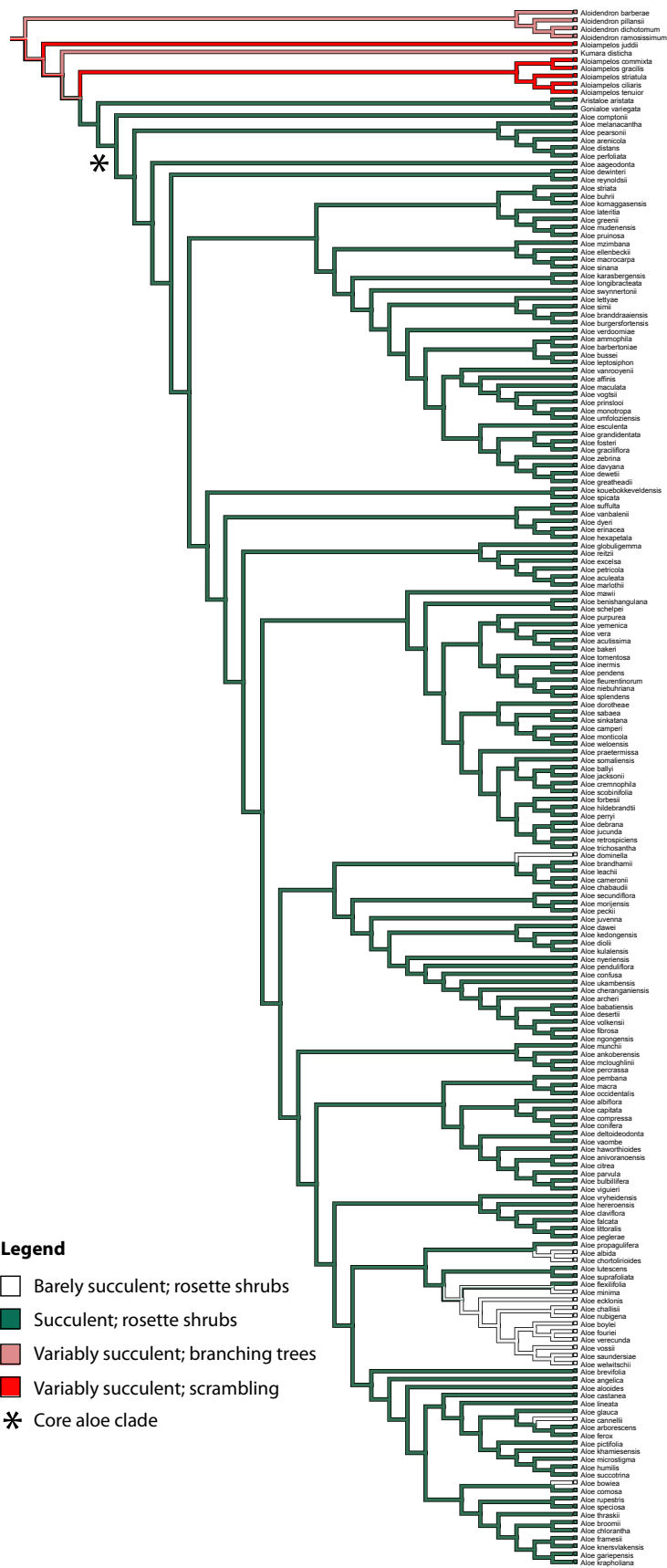

**b) Leaf succulence and habit**

Supplement: Additional file 5: — Phylogenetic distribution of leaf succulence, habit and medicinal use in alooid taxa. Most parsimonious reconstructions of character states mapped to Bayesian consensus tree in a) leaf succulence, b) habit and c) medicinal uses. [file 12862_2015_291_MOESM5_ESM.zip › 1692691512145080_add6.pdf]
