# Supplementary material for: Evolutionary history and leaf succulence as explanations for medicinal use in aloes and the global popularity of Aloe vera
Source: BMC Evol Biol. 2015 Feb 26;15:29. doi: 10.1186/s12862-015-0291-7 (PMC4342203; doi:10.1186/s12862-015-0291-7)
Supplement: Additional file 3: — Ancestral area reconstructions for Xanthorrhoeaceae subfamily Asphodeloideae. a) Ancestral areas displayed on the penalised likelihood-dated Bayesian consensus tree; b) detail of the clade containing Aloe vera. Legend refers to regions modified from [57] for this analysis: A, Southern Africa; B, Zambezi; C, Congolian; D, Ethiopian-Somalian; E, Saharan-Sudanian; F, Arabian; G, Madagascan; H, Eurasian; Trash, sum of ancestral area probabilities <0.1. [file 12862_2015_291_MOESM3_ESM.pdf]

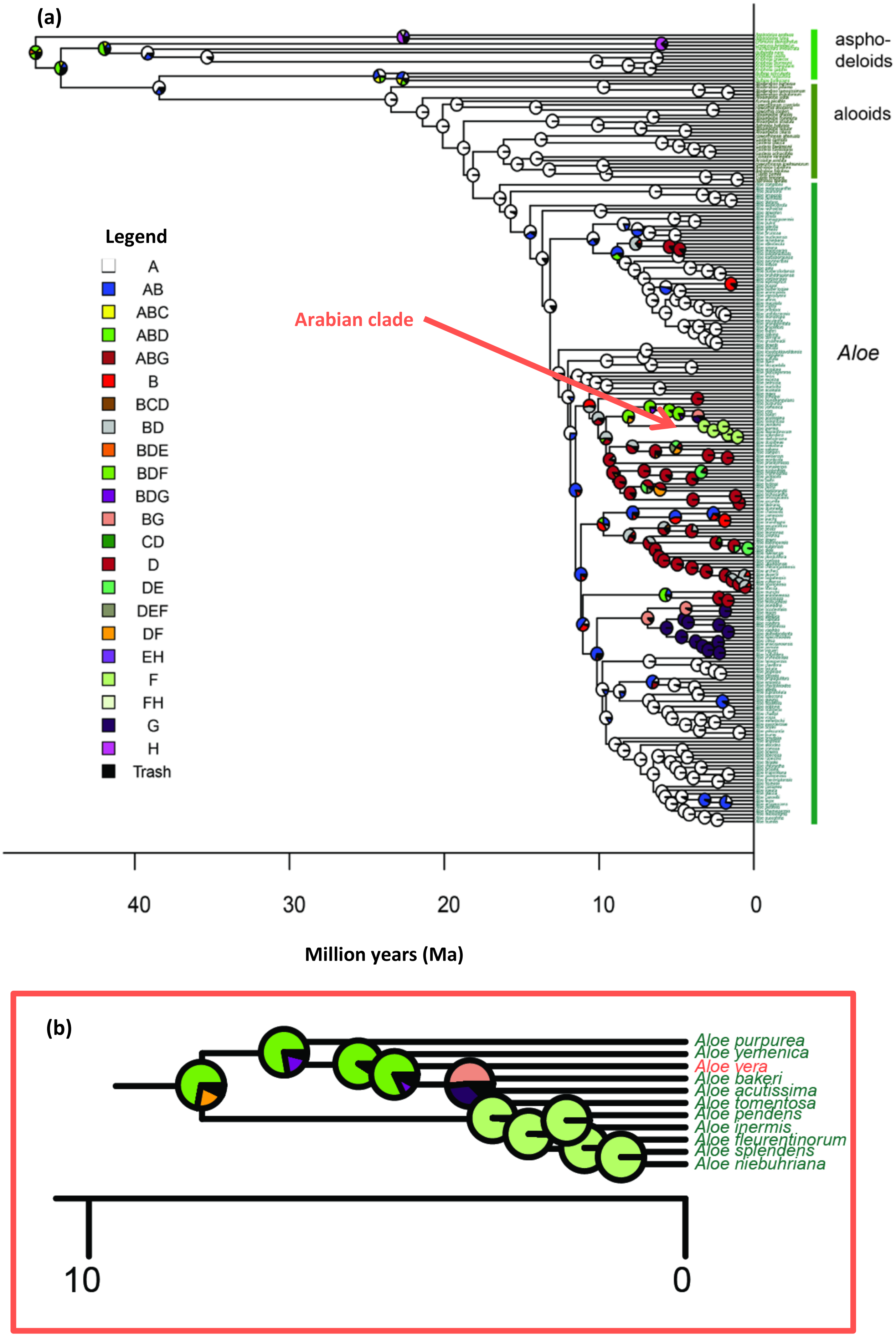

(a)
aspho-
deloids
alooids
Aloe
Legend
A
AB
ABC
ABD
ABG
B
BCD
BD
BDE
BDF
BDG
BG
CD
D
DE
DEF
DF
EH
F
FH
G
H
Trash
Arabian clade
40
30
20
10
0
Million years (Ma)
(b)
Aloe purpurea
Aloe yemenica
Aloe vera
Aloe bakeri
Aloe acutissima
Aloe tomentosa
Aloe pendens
Aloe inermis
Aloe fleurentinorum
Aloe splendens
Aloe niebuhriana
10
0

Ancestral area reconstructions for Xanthorrhoeaceae subfamily Asphodeloideae.
a) Ancestral areas displayed on the penalised likelihood-dated Bayesian consensus tree; b) detail of the clade containing *Aloe vera*. Legend refers to regions modified from (52) for this analysis: A, Southern Africa; B, Zambezi; C, Congolian; D, Ethiopian-Somalian; E, Saharan-Sudanian; F, Arabian; G, Madagascan; H, Eurasian; Trash, sum of ancestral area probabilities <0.1.
