# Supplementary material for: Evolutionary history and leaf succulence as explanations for medicinal use in aloes and the global popularity of Aloe vera
Source: BMC Evol Biol. 2015 Feb 26;15:29. doi: 10.1186/s12862-015-0291-7 (PMC4342203; doi:10.1186/s12862-015-0291-7)
Supplement: Additional file 5: — Phylogenetic distribution of leaf succulence, habit and medicinal use in alooid taxa. Most parsimonious reconstructions of character states mapped to Bayesian consensus tree in a) leaf succulence, b) habit and c) medicinal uses. [file 1692691512145080_add5.pdf › 1692691512145080_add5.pdf]

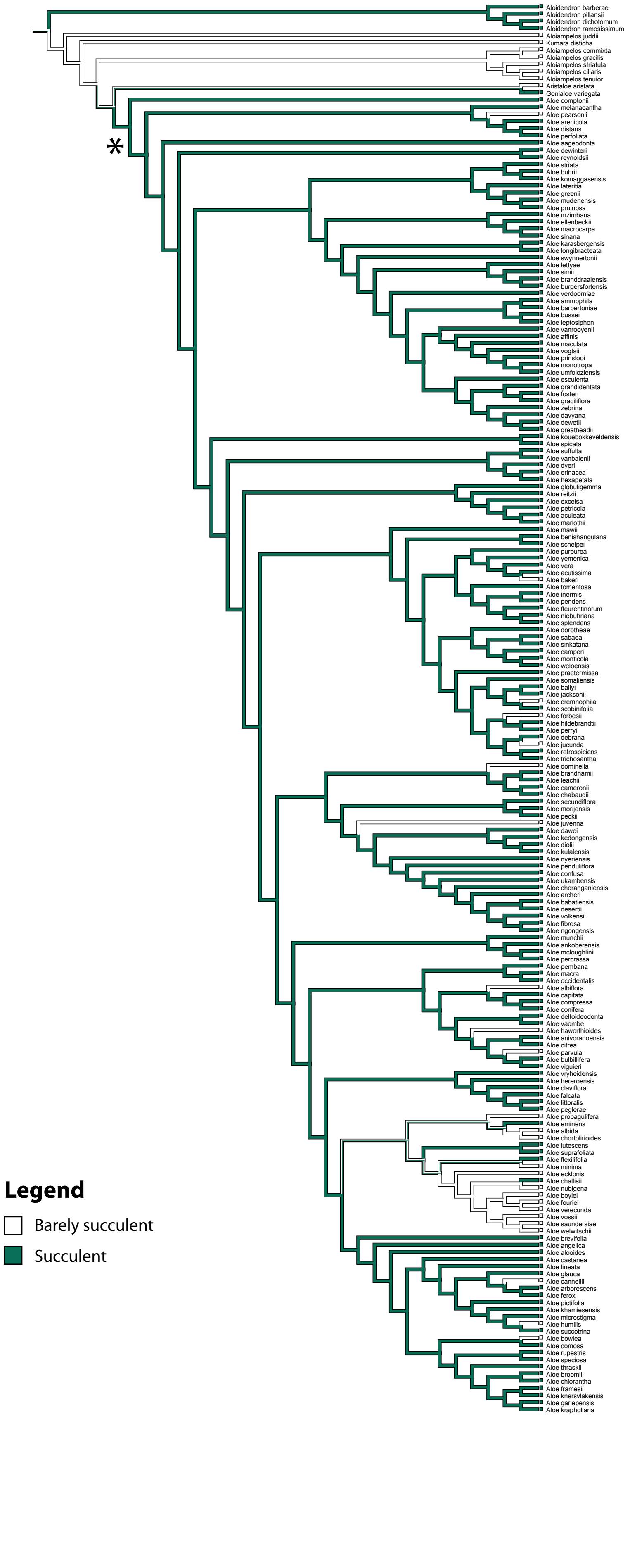
*
Aloidendron barberae
Aloidendron pillansii
Aloidendron dichotomum
Aloidendron ramosissimum
Aloiampelos juddii
Kumara disticha
Aloiampelos commixta
Aloiampelos gracilis
Aloiampelos striatula
Aloiampelos ciliaris
Aloiampelos tenuior
Aristaloe aristata
Gonialoe variegata
Aloe comptonii
Aloe melanacantha
Aloe pearsonii
Aloe arenicola
Aloe distans
Aloe perfoliata
Aloe aageodonta
Aloe dewinteri
Aloe reynoldsii
Aloe striata
Aloe buhrii
Aloe komaggasensis
Aloe lateritia
Aloe greenii
Aloe mudenensis
Aloe pruinosa
Aloe mzimbana
Aloe ellenbeckii
Aloe macrocarpa
Aloe sinana
Aloe karasbergensis
Aloe longibracteata
Aloe swynnertonii
Aloe lettyae
Aloe simii
Aloe branddraaiensis
Aloe burgersfortensis
Aloe verdoorniae
Aloe ammophila
Aloe barbertoniae
Aloe bussei
Aloe leptosiphon
Aloe vanrooyenii
Aloe affinis
Aloe maculata
Aloe vogtsii
Aloe prinslooi
Aloe monotropa
Aloe umfoloziensis
Aloe esculenta
Aloe grandidentata
Aloe fosteri
Aloe graciliflora
Aloe zebrina
Aloe davyana
Aloe dewetii
Aloe greatheadii
Aloe kouebokkeveldensis
Aloe spicata
Aloe suffulta
Aloe vanbalenii
Aloe dyeri
Aloe erinacea
Aloe hexapetala
Aloe globuligemma
Aloe reitzii
Aloe excelsa
Aloe petricola
Aloe aculeata
Aloe marlothii
Aloe mawii
Aloe benishangulana
Aloe schelpei
Aloe purpurea
Aloe yemenica
Aloe vera
Aloe acutissima
Aloe bakeri
Aloe tomentosa
Aloe inermis
Aloe pendens
Aloe fleurentinorum
Aloe niebuhriana
Aloe splendens
Aloe dorotheae
Aloe sabaea
Aloe sinkatana
Aloe camperi
Aloe monticola
Aloe weloensis
Aloe praetermissa
Aloe somaliensis
Aloe ballyi
Aloe jacksonii
Aloe cremnophila
Aloe scobinifolia
Aloe forbesii
Aloe hildebrandtii
Aloe perryi
Aloe debrana
Aloe jucunda
Aloe retrospiciens
Aloe trichosantha
Aloe dominella
Aloe brandhamii
Aloe leachii
Aloe cameronii
Aloe chabaudii
Aloe secundiflora
Aloe morijensis
Aloe peckii
Aloe juvenna
Aloe dawei
Aloe kedongensis
Aloe diolii
Aloe kulalensis
Aloe nyeriensis
Aloe penduliflora
Aloe confusa
Aloe ukambensis
Aloe cheranganiensis
Aloe archeri
Aloe babatiensis
Aloe desertii
Aloe volkensii
Aloe fibrosa
Aloe ngongensis
Aloe munchii
Aloe ankoberensis
Aloe mcloughlinii
Aloe percrassa
Aloe pembana
Aloe macra
Aloe occidentalis
Aloe albiflora
Aloe capitata
Aloe compressa
Aloe conifera
Aloe deltoideodonta
Aloe vaombe
Aloe haworthioides
Aloe anivoranoensis
Aloe citrea
Aloe parvula
Aloe bulbillifera
Aloe viguieri
Aloe vryheidensis
Aloe hereroensis
Aloe claviflora
Aloe falcata
Aloe littoralis
Aloe peglerae
Aloe propagulifera
Aloe eminens
Aloe albida
Aloe chortolirioides
Aloe lutescens
Aloe suprafoliata
Aloe flexilifolia
Aloe minima
Aloe ecklonis
Aloe challisii
Aloe nubigena
Aloe boylei
Aloe fouriei
Aloe verecunda
Aloe vossii
Aloe saundersiae
Aloe welwitschii
Aloe brevifolia
Aloe angelica
Aloe alooides
Aloe castanea
Aloe lineata
Aloe glauca
Aloe cannellii
Aloe arborescens
Aloe ferox
Aloe pictifolia
Aloe khamiesensis
Aloe microstigma
Aloe humilis
Aloe succotrina
Aloe bowiea
Aloe comosa
Aloe rupestris
Aloe speciosa
Aloe thraskii
Aloe broomii
Aloe chlorantha
Aloe framesii
Aloe knersvlakensis
Aloe gariepensis
Aloe krapholiana
Legend
Barely succulent
Succulent
