# Supplementary material for: Evolutionary history and leaf succulence as explanations for medicinal use in aloes and the global popularity of Aloe vera
Source: BMC Evol Biol. 2015 Feb 26;15:29. doi: 10.1186/s12862-015-0291-7 (PMC4342203; doi:10.1186/s12862-015-0291-7)
Supplement: Additional file 5: — Phylogenetic distribution of leaf succulence, habit and medicinal use in alooid taxa. Most parsimonious reconstructions of character states mapped to Bayesian consensus tree in a) leaf succulence, b) habit and c) medicinal uses. [file 1692691512145080_add7.pdf › 1692691512145080_add7.pdf]

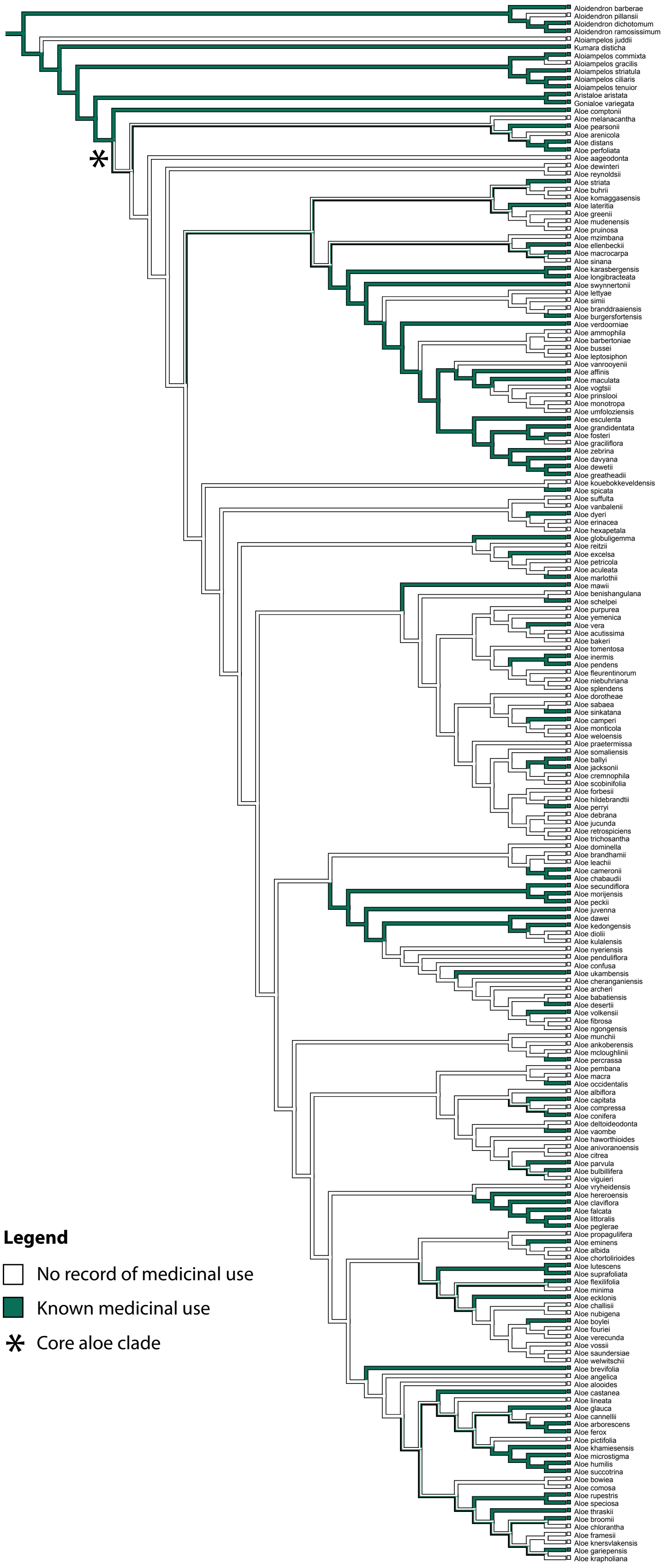

c) Medicinal use
