# Supplementary material for: Evolutionary history and leaf succulence as explanations for medicinal use in aloes and the global popularity of Aloe vera
Source: BMC Evol Biol. 2015 Feb 26;15:29. doi: 10.1186/s12862-015-0291-7 (PMC4342203; doi:10.1186/s12862-015-0291-7)
Supplement: Additional file 4: — Summary statistics for phylogenetic dataset. Taxon sampling, sequence length and model selection for data partitions. [file 12862_2015_291_MOESM4_ESM.pdf]

Additional file 4. Summary statistics for phylogenetic dataset.
Sampling, sequence length and model selection for data partitions.

	ITS	matK	psbA	rbcL	rps16	trnLF intron	trnLF spacer
Sequence alignment length	799	1622	765	1362	1041	1143	530
Number of taxa	175	207	64	92	159	55	55
Model selected by Akaike Information Criterion	HKY+G	GTR+G	HKY+I+G	GTR+I+G	HKY+G	HKY+G	HKY+G
